# Supplementary material for: Identification of photoperiod-induced specific miRNAs in the adrenal glands of Sunite sheep (Ovis aries)
Source: Front Vet Sci. 2022 Jul 22;9:888207. doi: 10.3389/fvets.2022.888207 (PMC9354845; doi:10.3389/fvets.2022.888207)
Supplement: Supplementary file 1 [file Data_Sheet_1.ZIP › Supplementary file/miRDeep2 file.zip!/miRDeep2 file/Novel_Structure/SP42A1/Novel_51.pdf]

Provisional ID : NC_019468.2_14807
Score total : 1.3
Score for star read(s) : -1.3
Score for read counts : 0
Score for mfe : 1
Score for randfold : 1.6
Score for cons. seed :
Total read count : 53
Mature read count : 53
Loop read count : 0
Star read count : 0

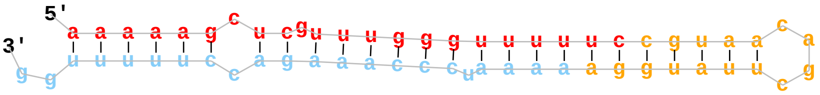

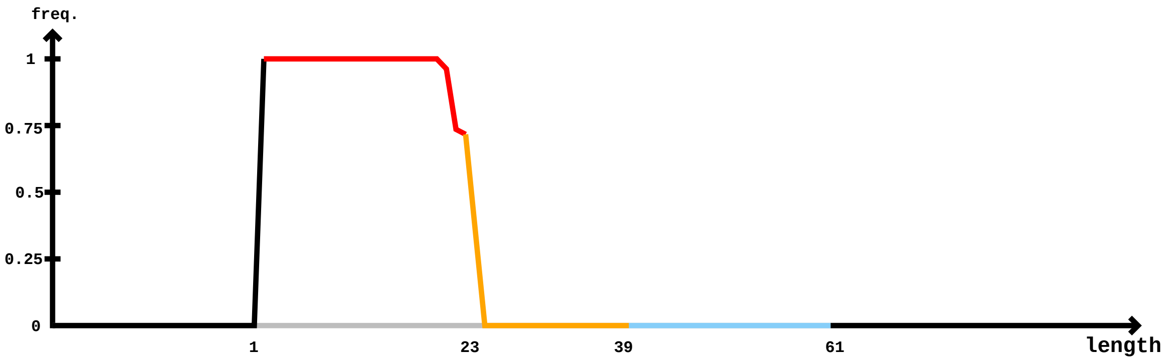

Mature Star

```
5'- cugcuccuguuggauugacaaaaaagcucguuuggguuuuuccguaacagcuuauggaaaaaucccaaagaccuuuuuggccaacccaauaaagaagaauuugaguuucau   -3'    exp
    .......(((((.(((.(.((((((.((.((((((((((((((((....))))))))))).))))))))).)))))).).))).))))))..((((........))))..    reads      mm        sample
    ....................aaaaagUucguuuggguuu.........................................................................    2          1         seq
    ....................aaaaagUucguuuggguuuu........................................................................    12         1         seq
    ....................aaaaagcucguuuggguuuuu.......................................................................    1          0         seq
    ....................aaaaagUucguuuggguuuuuc......................................................................    38         1         seq
```
